# Prevalence of past homelessness, mental health and health risk behaviours among sexual minority young people in the UK: insights from the Millennium Cohort Study

**DOI:** 10.1186/s12889-025-25429-7

**Published:** 2025-12-10

**Authors:** Amal R. Khanolkar, Laia Becares

**Affiliations:** 1 Department of Population Health Sciences, School of Life Course & Population Sciences, King’s College London, London, United Kingdom; 2 Department of Global Health and Social Medicine, King’s College London, London, United Kingdom

**Keywords:** Homelessness, Sexual minority, Mental health, Depression, Self-harm, Health-risk behaviour, Adolescent health, Health inequalities, United Kingdom

## Abstract

**Aim:**

Despite evidence on higher risk for homelessness and worse mental health in sexual minority (SM) adolescents, no study has examined these associations using nationally-representative data in the UK. This study examined inequalities in the prevalence of past homelessness by sexual identity, and whether rates of poor mental health and health-risk behaviours (HRBs) differ by sexual identity and past homelessness in adolescents.

**Methods:**

This cross-sectional study used data from the age 17 sweep of the UK-wide Millennium Cohort Study (*N* = 10,232, 51% female, 21% SM). Mental health was assessed using the Strengths and Difficulties Questionnaire (SDQ) emotional symptoms subscale for depression and anxiety, and self-reported actions of self-harm, attempted suicide, sleep quality and doctor-diagnosed depression. HRBs were measured using six variables, including regular smoking and antisocial behaviour. Associations between sexual identity (heterosexual, mainly heterosexual, bisexual and gay/lesbian) and past homelessness were examined using multivariable logistic regression. Associations between past homelessness and mental health and HRBs, and whether these differed by sexual identity were examined using multivariable logistic regression with appropriate interactions between the homeless and sexual identity variables. Models were adjusted for sex, parental income and ethnicity.

**Results:**

Bisexual (adjusted-OR 2.92, 95% CI: 1.47–5.82) and gay/lesbian (adjusted-OR 1.87, 0.53–6.56) adolescents had higher odds for past homelessness compared to heterosexual peers. Prevalence of self-harm (homeless bisexual/gay/lesbian and heterosexual: 85% and 39% respectively, not homeless bisexual/gay/lesbian and heterosexual: 55% and 18% respectively) and attempted suicide (homeless bisexual/gay/lesbian and heterosexual: 52% and 22% respectively, not homeless bisexual/gay/lesbian and heterosexual: 18% and 5% respectively) were significantly higher in individuals with past homelessness with the highest rates among SM youth. Similar pattern of findings was observed with regular smoking and antisocial behaviour.

**Conclusions:**

SM individuals are more likely to experience past homelessness. Past experiences of homelessness are associated with worse current mental health and HRBs regardless of sexual orientation. However, greater proportions of SM adolescents with past experiences of homelessness reported worse mental health and some HRBs compared to heterosexual peers with the same experience.

**Supplementary Information:**

The online version contains supplementary material available at 10.1186/s12889-025-25429-7.

## Introduction

Homelessness among young people is a serious social and public health concern. Experiences of homelessness are associated with significant short and long-term adverse health and social problems, including increased likelihood for a range of sustained and recurrent mental health problems, substance use, experiences of violence, sexual exploitation, victimisation and discrimination, poor self-rated general health and quality of life, and reduced access to continuing and higher education [1–4]. Experiencing homelessness is considered an indicator of extreme social exclusion and adversity [5]. In the UK, there is no official government database on the prevalence of homelessness among young people, and the groups most impacted by it. *Centre Point*, a national charity working on combating youth homelessness which carries out the largest annual country-wide survey on homelessness, found that 118,134 young people aged 16–24 years were homeless in 2023-24 (a 10% increase from preceding years) [6]. This number is likely to be an underestimate as it only includes young people who approached local councils for help [6]. The most common reasons cited for youth homelessness includ family disputes and domestic violence, evictions, and no longer being supported by family or friends [6]. Homelessness disproportionately affects vulnerable groups including immigrants and refugees, those from socioeconomically disadvantaged backgrounds, single-parent households, and sexual minority groups [7–9].

A growing body of evidence, primarily from North America and to a lesser extent Australia, indicates that homelessness is disproportionately experienced by sexual minority (SM) youth compared to heterosexual peers [8, 10, 11]. Quantitative studies (using different designs and both probability and non-probability samples) indicate prevalence estimates of 15 to 45% of homeless respondents identify as sexual and/or gender minority (lesbian, gay, bisexual, trans, queer and other sexual/gender minority identities or LGBTQ+) [8]. However, estimates from empirical studies with heterosexual comparator groups are limited in number and generalisability as the proportion of individuals identifying as SM has increased substantially in recent years. Further, SM youth make-up a large proportion (nearly 40%) of the clientele served by homelessness agencies and charities across the USA (whereas the proportion of individuals identifying as SM in the general population is 5 to 15%) [12]. A review of the literature by Ecker showed that prevalence rates of homelessness among SM youth ranges from 13% to 37% with the majority falling around the range of 20–35% [8, 13]. One of the largest studies to date examining prevalence of homelessness by sexual identity in the USA (including 28,405 SM and 136,232 heterosexual individuals) found 12% of SM youth experienced homelessness compared to 4% among heterosexual peers [14]. Thus, regardless of sample size, country of study, whether all SM subgroups are combined or examined separately, this group consistently has disproportionately higher rates of homelessness than heterosexual peers.

Studies have also shown that the prevalence of mental health problems (such as depression, suicidality, and to lesser extent sleep quality) and health-risk behaviours ([HRBs], including substance use) are significantly higher among homeless SM youth compared to homeless heterosexual and non-homeless SM peers [15, 16]. Homeless SM youth are also more likely to engage in risky sexual behaviour as a means for survival or due to sexual exploitation [8, 17].

The reasons for increased rates of homelessness and higher prevalence of mental health problems among SM people include family conflict and rejection, victimisation, abuse, and being told to leave home due to *‘coming out’* or disclosing of SM orientation [17–21]. Other factors include the higher levels of childhood abuse and neglect, homophobic bullying (both within and external to the familial environment) and higher levels of mental health problems among SM youth compared to heterosexual peers [20, 22, 23]. Although breakdown of relationships with family, experiences of abuse in childhood, poverty and socioeconomic disadvantaged backgrounds increase risk of homelessness among both SM and non-SM youth [8], these factors are particularly deleterious for SM youth, who experience rejection because of their sexual identity [17]. Further, many of these risk factors are likely to co-occur leading to higher risk for homelessness among SM youth compared to heterosexual peers.

Despite concerning prevalence of both homelessness and mental health problems among SM people, the basic epidemiology of homelessness experiences among UK SM youth and impact on health and HRBs is largely not known. This prevents any meaningful discussion, further research and policy development. The reason for this gap in evidence and practice is that larger scale and national studies on prevalence of homelessness among SM groups *in comparison* to heterosexual peers are not available in the UK [8]. The only larger-scale studies in the UK that reported on prevalence of homelessness are online surveys that included self-selecting SM individuals only or based on smaller regional/single cities only [24]. A recent UK-wide survey including 1119 LGBTQ + individuals of all ages, found that 20% had experienced homelessness [24]. In fact, most of the existing evidence on homelessness in SM youth in the UK including prevalence, common causes and health have been conducted by charities working exclusively with SM youth homelessness [8]. The lack of robust comparative studies using data from the UK general population has been cited repeatedly as a cause for concern [8, 25].

This study aims to address these gaps by examining associations between sexual identity and past homelessness among youth, including differences by sexual identity sub-group. Further, we aim to examine whether past homelessness is associated with worse mental health and higher levels of HRBs in SM youth compared to heterosexual peers with and without homelessness.

## Methods

### Study design and participants

This cross-sectional study utilised data from the age 17 sweep of the Millennium Cohort Study (MCS), a UK birth cohort study following the lives of 19,519 children born at the start of the millennium (2000–2002) [26]. Study participants have been followed over seven sweeps to date (9 months, 3, 5, 7, 11, 14 and 17 years). Detailed information on the study, sampling and survey design can be found at: https://cls.ucl.ac.uk/cls-studies/millennium-cohort-study/. 14,496 families were invited to participate in the age 17 sweep. Of this number, 10,625 (73.3%) families and 10,345 (71.4%) adolescents were successfully interviewed. This study included *N* = 10,232 children (98.9% of the eligible sample) who attended the age 17 sweep in 2018–2019. MCS was oversampled to include higher proportions of ethnic minority (EM) and socioeconomically disadvantaged families (sample weights ensure national representativeness). Attrition at the age 17 sweep was predicted by single-parent families, lower-income occupation and lower educational level, Black ethnicity, and male sex. The STrengthening the Reporting of OBservational studies in Epidemiology (STROBE) were followed in writing this manuscript.

### Measures

Sexual identity: Participants were asked about their current sexual identity (‘’*Which of the following options best describes how you currently think of yourself?*’’ with eight possible options to choose from and listed in Supplementary Table 1) Based on self-reported answers on sexuality, participants were categorized into: (1) Completely heterosexual, (2) Mainly heterosexual, (3) Bisexual and (4) Gay or lesbian. Those who answered ‘other’, ‘do not know’ and ‘preferred not to say’ were excluded (*N* = 154). We created a second sexual identity variable (combining bisexual and gay/lesbian individuals due to small numbers) with three categories: (1) Completely heterosexual, (2) Mainly heterosexual and (3) Bisexual, gay and lesbian (SM sub-groups combined).

#### Homelessness

Past reported experiences of homelessness (anytime previously) was a binary variable assessed by a single question *‘’Has there ever been a time when you were homeless with no permanent residence?”* Options to respond were yes or no.

#### Mental health (MH)

MH at age 17 was assessed using multiple indicators, all being self-reported (see Supplemental Table 2 for details). The Strengths and Difficulties Questionnaire (SDQ) is a commonly and widely used brief screening tool comprising 5 subscales (with each subscale including 5 items for a total of 25 items) that assess a range of behavioural and emotional problems in children [27]. We used the SDQ emotional symptoms subscale (SDQ-E) comprising 5 items or questions (e.g., *are you often unhappy?*) relating to depression, worry, fear, nervousness, and somatic symptoms, which assesses symptoms of psychological distress in the preceding six months. The SDQ-E is widely validated and *routinely used to capture symptoms of depression and anxiety* in the general population and clinical settings [28]. Further, the SDQ-E measure has been validated against diagnostic measures of depression or anxiety disorders and is an established screening tool to distinguish those individuals who meet the diagnostic criteria for depression and anxiety from those who do not. For each of the 5 items, participants could respond with: Not true, somewhat true, or certainly true (scored 0, 1 and 2, respectively). Total scores were calculated, ranging from 0 to 10, with higher scores indicating more emotional problems (symptoms of depression and anxiety). Total scores were categorised into a binary variable based on recommended cut-off points indicating participants with ‘close to average’ (< 6) vs. ‘high/very high levels’ (≥ 6) of difficulties [29].

Other mental health indicators included lifetime doctor diagnosed depression (yes vs. no), self-harm in the previous one year (actions like burning, bruising/pinching, taking an overdose of tablets and pulling out hair), and lifetime attempted suicide (yes vs. no). Participants were asked about quality of sleep in the in the previous month and could choose from one of four options (very good, fairly good, fairly bad or very bad). Categories were combined to create a binary variable; very good/fairly good vs. fairly bad/very bad sleep. Social adversity was assessed by experiences of victimisation (i.e., experiences of verbal, physical, sexual assault and/or harassment in the past 12 months). Responses were categorised into no experiences vs. yes.

General health was assessed by the question *‘’How would you describe your health generally?’’* with possible answers being excellent, very good, good, fair, or poor. Responses were categorised into a binary variable (excellent, very good, or good vs. fair or poor).

#### Health-risk behaviours (HRBs)

 All HRBs at age 17 are self-reported and were coded as binary indicators (no vs. yes *or* low vs. high frequencies). HRBs were six in number and included regular current smoking habits (ever smokers and current smokers at age 17), frequency of alcohol consumption (< 10 times vs. ≥10 times) in previous 12 months, and frequency of cannabis use (≤ 4 times vs. ≥5times) in previous 12 months. These frequencies were chosen based on prior research using the same variables in relation to sexual identity health and ensuring sufficient statistical power [22]. Participants were asked about regular use of contraception or protection during sex with partners (lifetime sexual activity without use of any type of contraception or protection). Answers were categorised as none vs. yes. We also examined frequency of physical activity in the previous week (none vs. any). Antisocial behaviour was assessed by one or more of the following acts in the previous 12 months: Pushed or shoved/hit/slapped/punched someone, hit someone with or used a weapon, stolen something from someone, harassed someone via mobile phone/email, sent pictures or spread rumours about someone and made unwelcome sexual approaches/sexually assaulted someone. The original questions, complete component items of each scale and all health-related indicators, and how they were categorised (including references) are listed in complete detail in Supplementary Table 2.

#### Covariates

These included parental income, ethnicity and sex assigned at birth (hereafter sex). Parental income was used as an indicator of socioeconomic position ascertained at age 3. Household income (Organisation for Economic Co-operation and Development UK) was categorized into equalized quintiles (where quintiles 1 and 5 represent the lowest and highest income quintiles respectively). For analysis, quintiles 4 and 5 were combined into a single category. Parents (or guardians) reported participant’s ethnicity at age 3 (original categories in Supplemental Table 1) which was available for over 95% of participants. Information on ethnicity from subsequent sweeps (ages 7, 11 and 14) was used to replace any missing ethnicity at age 3. Subjects were grouped into either (1) White (ethnic majority) and (2) EM (mixed-ethnicity, South Asian, Black and ‘other’ EM groups). The ‘other’ group included participants from Asia (excluding South Asia), the Middle East and South America.

#### Missing data

Due to attrition over follow-up, the proportion of missingness varied from 4.8% to 36%. Missing data for all health indicators and HRBs was < 5% and data were largely complete for sociodemographic variables (< 0.5% missing for parental income and educational level but 4.3% were missing data on sexual identity), and all participants had complete data on sex and ethnicity. Missing data was addressed using Multiple Imputation by Chained Equations (MICE), operating under the Missing at Random Assumption (MAR). This was suitable given that observed characteristics like ethnicity and parental income were independent predictors of missingness (i.e., ethnic minority and socioeconomically disadvantaged participants were more likely to have missing information across ≥ 1 analysis parameters). To predict missing data, the imputation model integrated all mental health indicators, HRBs, homelessness, and all covariates. The model also included auxiliary variables like mental wellbeing indicators, maternal age at birth and parental highest educational qualifications. We also included interaction terms between homelessness and sexual identity variables. Estimates were obtained by pooling results across 35 imputed data sets which were combined using Rubin’s rules. Further, comparison of the results obtained from the complete cases and imputed analysis showed that the standard errors were smaller for multiple imputation, demonstrating the efficiency gain of multiple imputation over complete cases analysis. This helped to avoid bias in the resulting estimates, which could have been attributed to the disproportionate contribution of more socioeconomically advantaged participants (potentially leading to underestimation of sexual identity-homelessness associations). The imputed sample was used in all analyses.

### Statistical analysis

We first examined associations between sexual identity and risk for past homelessness using logistic regression modelling. Models were run unadjusted and adjusted for sex, parental income and ethnicity.

To examine whether SM individuals with past homelessness were more likely to report worse mental and general health compared to heterosexual peers with and without past homelessness, we ran multivariable logistic regression models with interaction terms between the sexual identity and homelessness variables. These models were adjusted for sex, parental income, and ethnicity. Similarly, we also ran multivariable logistic models to examine whether HRBs were more likely to be reported by SM individuals with past homelessness and compared to heterosexual peers (adjusted for confounders as above). The second version of the sexual identity variable was used in these models due to the overall smaller number of individuals reporting past homelessness. The predicted probabilities for all outcomes across the combinations of sexual identity and past homelessness indicators (i.e., six categories generated by the interaction terms between the two variables, e.g., heterosexual and homeless) were estimated for all logistic regression models using the *’margins’* command in Stata. This generates adjusted predictive margins or the probability for each outcome in each group of interest. Predicted probabilities were visualised (by creating interaction plots using the *‘marginsplot’* command) to aid understanding of the interaction terms between the homeless and sexual identity variables.

All models were weighted with non-response weights from the birth sweep to account for the stratified cluster design of the MCS and attrition over time (using the Stata *‘svy’* command for survey data). Odds ratios from models were plotted for visualisation. All analyses were conducted in Stata V18 (StataCorp LP, College Station, Texas).

## Results

Table 1 displays the proportions of sexual identity, all health outcomes, and HRBs by past experiences of homelessness. Of the 10,232 adolescents in this study, 91 individuals reported past experiences of homelessness with significant variation in proportions by sexual identity (0.9% among heterosexuals, compared to 2.4% in bisexuals and 1.3% in gay/lesbian individuals, *p* < 0.005). Adolescents reporting past experiences of homelessness had higher prevalence of all mental and general health outcomes at age 17 (e.g., 4% vs. 0.7% for attempted suicide and poor general health, 2% vs. 0.6% for self-harm among those with and without past homelessness, respectively). Adolescents with past experiences of homelessness were more likely to report regular smoking and sex without contraception but there were no differences in frequent alcohol and cannabis use, exercise or anti-social behaviour.

**Table 1:** Characteristics of adolescents (*N* = 10,232) aged 17 years from the Millennium Cohort Study reporting adverse health and health risk behaviours by past experiences of homelessness

	Past homelessness					
	No			Yes		
	%	95% CI		%	95% CI	
	99.1	98.9	99.3	0.94	0.75	
**Sexual identity**						
Heterosexual	99.1	98.5	99.7	0.9	0.3	1.5
Mainly heterosexual	99.2	99.0	99.4	0.8	0.6	1.0
Bisexual	97.6	96.5	98.8	2.4	1.2	3.5
Gay/lesbian	98.7	97.2	100.1	1.3	−0.1	2.8
**Health indicator**s						
SDQ emotional symptoms subscale: total scores: ≥6	0.7	0.5	0.9	2	1	2
Doctor diagnosed depression	0.7	0.5	0.8	3	2	4
Self-harm (past year)	0.6	0.5	0.8	2	1	2
Attempted suicide (lifetime)	0.7	0.5	0.9	4	3	5
Poor sleep quality	0.6	0.4	0.9	2	1	2
Poor general health	0.7	0.5	0.9	4	2	5
Health risk behaviours						
Current regular smoking	0.7	0.5	0.9	3	2	4
Alcohol frequency (≥ 10 times in past year)	1	0.8	1	0.7	0.3	1
Cannabis frequency (≥ 5times in past year)	0.9	0.7	1	2	0.6	2
No exercise (in previous week)	0.8	0.6	1	1	0.8	2
Sex without contraception (regular use)	0.8	0.6	0.9	2	1	2
Antisocial behaviour (past year)	0.9	0.7	1.2	1	0.7	1.4
Victimisation	0.6	0.4	0.8	1.3	1	1.7
**Ethnicity**						
White	99.1	98.9	99.3	0.9	0.7	1.1
Non-White	98.9	98.5	99.4	1.1	0.59	1.5

Table 2 displays proportions of all outcomes in relation to intersectional sexual identity and past homelessness. SM adolescents with past experiences of homelessness consistently had higher proportions of individuals reporting worse mental health (observed for all indicators) compared to heterosexual and SM peers with and without past homelessness, respectively. For example, 55.6% (95% CI 32.6–78.5) of SM adolescents with past experiences of homelessness reported symptoms of depression and anxiety, while corresponding proportions were 37% (25.1–49.0) in heterosexuals with past homelessness, and 47% (43.6–50.3) and 18.2% (17.3–19.0) in SM and heterosexual peers without past homelessness, respectively. A similar trend was observed for regular smoking and sex without contraception.

**Table 2:** Proportions of adolescents (*N* = 10,232) from the Millennium Cohort Study reporting adverse health and health risk behaviours at age 17 years by past experiences of homelessness and sexual identity

Sexual identity & past homelessness experience	SDQ emotional symptoms subscale^a^			Doctor diagnosed depression			Self-harm^b^			Attempted suicide^c^			Poor sleep quality^d^			Poor general health		
	%	95% CI		%	95% CI		%	95% CI		%	95% CI		%	95% CI		%	95% CI	
Not homeless & heterosexual	18.2	17.3	19.0	7.9	7.3	8.5	18.2	17.3	19.0	5.7	5.2	6.2	29.7	28.3	31.0	6.1	5.6	6.7
Not homeless & mainly heterosexual	32.5	29.6	35.3	14.4	12.3	16.6	36.3	33.4	39.3	9.1	7.3	10.8	38.7	35.4	42.0	9.2	7.4	10.9
Not homeless & bisexual/gay/lesbian	47.0	43.6	50.3	26.4	23.4	29.4	57.3	54.0	60.6	21.6	18.8	24.3	44.7	40.8	48.6	12.2	10.0	14.4
Homeless & heterosexual	37.0	25.1	49.0	26.0	15.1	36.9	35.9	24.1	47.7	22.7	12.3	33.1	51.9	36.6	67.1	25.0	14.4	35.6
Homeless & mainly heterosexual	44.4	12.0	76.9	55.6	23.1	88.0	51.4	17.2	85.7	11.1	−9.4	31.6	34.3	−2.7	71.3	55.6	23.1	88.0
Homeless & bisexual/gay/lesbian	55.6	32.6	78.5	61.1	38.6	83.6	88.9	74.4	103.4	77.8	58.6	97.0	75.1	49.2	100.9	33.3	11.6	55.1

	Current regular smoking			Alcohol frequency ≥10 times^b^			Cannabis frequency > 4times ^b^			No exercise ^e^			Sex without contraception ^f^			Antisocial behaviour ^b^		
	%	95% CI		%	95% CI		%	95% CI		%	95% CI		%	95% CI		%	95% CI	
Not homeless & heterosexual	10.7	10.0	11.3	20.0	19.1	20.9	7.5	6.9	8.0	23.1	22.2	24.0	17.2	16.4	18.1	25.4	24.5	26.4
Not homeless & mainly heterosexual	10.7	8.8	12.5	24.1	21.5	26.7	11.1	9.2	13.0	26.8	24.1	29.5	19.0	16.6	21.4	28.8	26.1	31.6
Not Homeless & gay/lesbian	16.2	13.8	18.7	22.7	19.9	25.5	10.0	8.0	12.0	32.6	29.5	35.7	23.3	20.5	26.1	26.2	23.2	29.1
Homeless & heterosexual	29.2	18.0	40.5	10.1	2.5	17.7	12.1	3.9	20.4	32.8	21.3	44.3	26.3	15.4	37.3	20.0	10.1	30.0
Homeless & mainly heterosexual	22.2	−4.9	49.4	22.2	−4.9	49.4	13.0	−10.4	36.4	22.2	−4.9	49.4	33.3	2.5	64.1	55.6	23.1	88.0
Homeless & bisexual/gay/lesbian	55.6	32.6	78.5	27.8	7.1	48.5	16.7	−0.6	33.9	38.9	16.4	61.4	50.0	26.9	73.1	50.0	26.9	73.1

*SDQ:* Strengths & difficulties questionnaire

^a^Those with scores ≥ 6 (high/very high levels of emotional difficulties)

^b^in previous year/12 months

^c^lifetime attempted suicide

^d^sleep quality in previous month

^e^in previous week

^f^regular use of contraception or protection

### Sexual identity differences in past homelessness

Compared to heterosexual adolescents, SM individuals were more likely to report past experiences of homelessness, with findings statistically significant in bisexual individuals (adjusted odds ratio [OR] 2.92, 1.47–5.82). Gay/lesbian individuals were also more likely to report past homelessness, though findings were not statistically significant (adjusted OR 1.87, 0.53–6.56 for, Table 3). Adjustment for covariates only marginally attenuated the estimates.

**Table 3:** Associations between sexual identity and past experiences of homelessness in 10,232 adolescents aged 17 years from the millennium cohort Study. Estimates are from logistic regression models

Predictor	Unadjusted model		Adjusted model^a^	
	OR	95% CI	OR	95% CI
Sexual identity				
Heterosexual	1		1	
Mainly heterosexual	1.04	0.48, 2.27	1.12	0.51, 2.49
Bisexual	**3.09**	**1.59, 6.00**	**2.92**	**1.47, 5.82**
Gay/lesbian	1.93	0.55, 6.75	1.87	0.53, 6.56

Estimates in bold indicate 95% CIs that do not include 1. For estimates of covariates, see Supplemental Table 3

^a^Adjusted for sex assigned at birth, ethnicity and parental income

### Differences in health and HRBs based on sexual identity and past homelessness

**Fig. 1:**
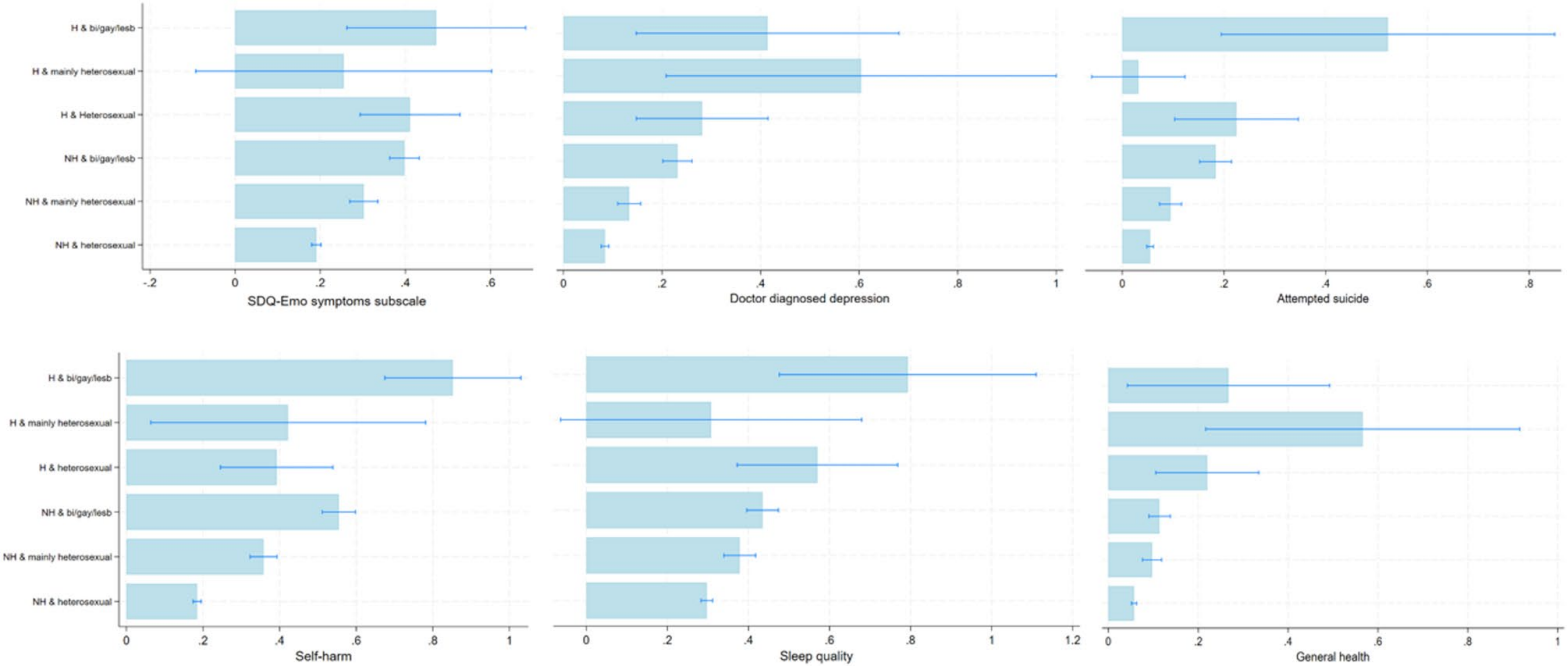
Predicted probabilities for health outcomes in 10,232 adolescents aged 17 years from the Millennium Cohort Study based on past experiences of homelessness and sexual identity. Estimates are based on multivariable logistic regression models (Supplemental Table 4) Note: H = Past homelessness NH = No past homelessness

**Fig. 2:**
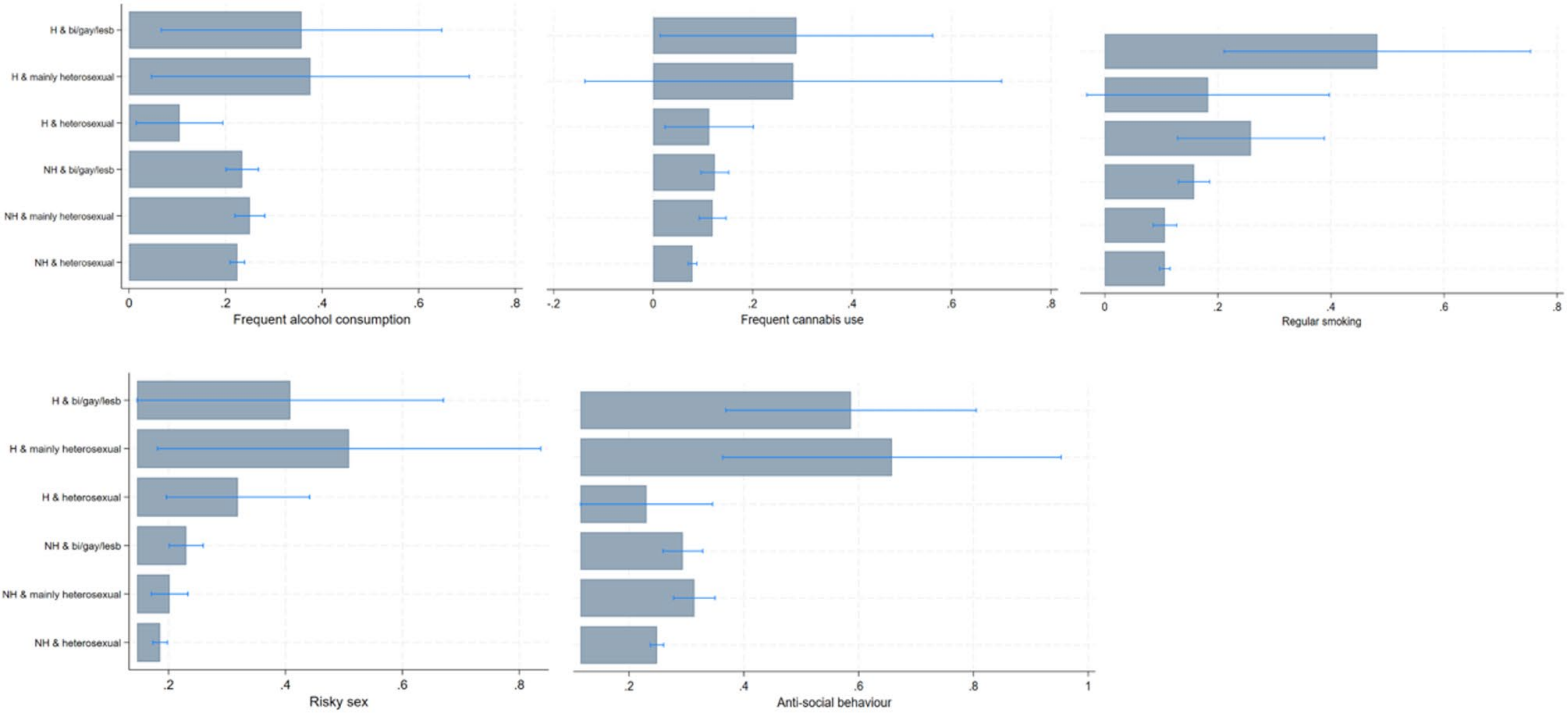
Predicted probabilities for health risk behaviours in 10,232 adolescents aged 17 years from the Millennium Cohort Study based on past experiences of homelessness and sexual identity. Estimates are based on multivariable logistic regression models (Supplemental Table 5) Note: H = Past homelessness NH = No past homelessness

In general, we found that adolescents with past experiences of homelessness had substantially higher proportions reporting worse health compared to those without homelessness. But these differences also varied by sexual identity and were often higher among SM adolescents with past experiences of homelessness and compared to SM peers without homelessness (see predicted probabilities in Table 4; Figs. 1 and 2, and Supplemental Tables 3 and 4). For example, on average, 18% (17–19) of heterosexual adolescents without past experiences of homelessness reported self-harm which increased to 39% (25–54) in heterosexuals with past experiences of homelessness. Corresponding proportions of self-harm were 55% (51–60) and 85% (67–99) in SM adolescents without and with past experiences of homelessness, respectively. Similarly, among adolescents without past homelessness, 6% (5–6), 10% (8–12) and 11% (9–14) of heterosexual, mainly heterosexual and SM adolescents, respectively, reported poor general health. This increased to 22% (11–33), 57% (22–92) and 27% (4–49) among corresponding sexual identity groups *with* past experiences of homelessness, respectively. We observed similar patterns for doctor diagnosed depression and poor sleep. However, differences in proportions were less pronounced among mainly heterosexual individuals for all outcomes. In general, despite the wider confidence intervals observed for groups with past experiences of homelessness, there was a consistent pattern for higher probabilities for worse mental health in SM and heterosexual adolescents with past homelessness, compared to peers without homelessness.

**Table 4:** Estimated probabilities for health and health risk behaviours in 10,232 adolescents aged 17 years from the millennium cohort study based on sexual identity and past experiences of homelessness. Estimates are derived from multivariable logistic regression models

Sexual identity & past homelessness	SDQ Emotional symptoms subscale^a^		Doctor diagnosed depression		Self-harm^b^		Attempted suicide^c^		Poor general health		Poor sleep quality^d^	
	Prob	95% CI	Prob	95% CI	Prob	95% CI	Prob	95% CI	Prob	95% CI	Prob	95% CI
Not homeless & Heterosexual	0.19	0.18–0.20	0.08	0.08–0.10	0.18	0.17–0.19	0.05	0.05–0.06	0.06	0.05–0.06	0.30	0.28–0.31
Not homeless & mainly heterosexual	0.30	0.27–0.33	0.13	0.11–0.20	0.36	0.32–0.39	0.09	0.07–0.12	0.10	0.08–0.12	0.38	0.34–0.42
Not homeless & bisexual/gay/lesbian	0.40	0.36–0.43	0.23	0.20–0.30	0.55	0.51–0.60	0.18	0.15–0.21	0.11	0.09–0.14	0.43	0.40–0.47
Homeless & Heterosexual	0.41	0.29–0.53	0.28	0.15–0.40	0.39	0.25–0.54	0.22	0.10–0.35	0.22	0.11–0.33	0.57	0.37–0.77
Homeless & mainly heterosexual	0.26	−0.10,0.60	0.60	0.21–1.00	0.42	0.06–0.78	0.03	−0.06,0.12	0.57	0.22–0.92	0.31	−0.06, 0.68
Homeless & bisexual/gay/lesbian	0.47	0.26–0.68	0.41	0.15–0.70	0.85	0.67–0.99	0.52	0.19–0.85	0.27	0.04–0.49	0.79	0.47–1.12

	**Alcohol frequency** (≥ 10 times^b^)		**Cannabis frequency** **(> 4 times**^**b**^**)**		**Current regular smoking**		**Sex without contraception**^**e**^		**Anti-social behaviour**^**b**^			
	**Prob**	**95% CI**	**Prob**	**95% CI**	**Prob**	**95% CI**	**Prob**	**95% CI**	**Prob**	**95% CI**		
Not homeless & Heterosexual	0.22	0.21–0.24	0.08	0.07–0.1	0.11	0.10–0.12	0.19	0.17–0.20	0.25	0.24–0.26		
Not homeless & mainly heterosexual	0.25	0.22–0.28	0.12	0.09–0.20	0.11	0.09–0.13	0.20	0.17–0.23	0.31	0.28–0.35		
Not homeless & bisexual/gay/lesbian	0.23	0.20–0.27	0.12	0.10–0.20	0.16	0.13–0.19	0.23	0.20–0.26	0.29	0.26–0.33		
Homeless & Heterosexual	0.11	0.02–0.20	0.11	0.02–0.20	0.26	0.13–0.40	0.32	0.20–0.45	0.23	0.12–0.35		
Homeless & mainly heterosexual	0.38	0.05–0.70	0.26	−0.10,0.7	0.18	−0.10,0.39	0.51	0.18–0.83	0.66	0.36–0.95		
Homeless & bisexual/gay/lesbian	0.36	0.07–0.65	0.29	0.01–0.6	0.48	0.21–0.75	0.41	0.14–0.67	0.58	0.36–0.80		

Estimates are derived from multivariable logistic regression models including interaction terms between homelessness and sexual identity variables and adjusted for sex assigned at birth, ethnicity and parental income

*SDQ:* Strengths & difficulties questionnaire

^a^Those with scores ≥ 6 (high/very high levels of emotional difficulties)

^b^in previous year/12 months

^c^lifetime attempted suicide

^d^sleep quality in previous month

^e^regular use of contraception or protection

Differences in proportions of adolescents reporting HRBs in relation to sexual identity and homelessness were less pronounced. However, there were observable differences for current regular smoking (11% [10-12] vs. 26% [13–40] in heterosexual, 11% [9-13] vs 18% [10-39] in mainly heterosexual, and 16% [13-19] vs. 48% [21-75] in SM adolescents) without and with homelessness, respectively. We found a similar pattern for sex without contraception (19% [17-20] 20% [17-23] vs. 51% [18–83]% in mainly heterosexual, 23% [20-26] vs. 41% [14–67] in SM adolescents) without and with homelessness, respectively. Similarly, significantly greater proportions of mainly heterosexual and SM adolescents with past homelessness reported anti-social behaviour compared to heterosexual peers with past homelessness. But there were no differences in anti-social behaviour among heterosexual peers (see Table 4; Fig. 2, and Supplemental Table 5).

## Discussion

In this study of adolescents in a UK national cohort study, we found that sexual minority adolescents aged 17 years were more likely to report past experiences of homelessness compared to heterosexual peers. While these findings were statistically significant in only bisexual adolescents, there was strong indication that gay/lesbian peers were also more likely to report past homelessness compared the heterosexual adolescents. We found that adolescents with past experiences of homelessness were substantially more likely to report worse mental and general health at age 17 compared to those without homelessness. However, findings suggest greater proportions of sexual minority individuals with past experiences of homelessness reported worse mental health (e.g., self-harm) compared to heterosexual peers with the same experience. Similar patterns in relation to sexual identity and past homelessness were observed for some HRBs like anti-social behaviour.

### Strengths and limitations

Strengths of this study include its sample, drawn from a nationally representative, contemporary birth cohort. This facilitates generalisability to the UK adolescent population and addressed a gap in evidence that has over relied on convenience samples. This study included multiple and widely used mental health indicators (like SDQ, self-harm, attempted suicide) which showed consistent associations with past homelessness status, reducing risk of chance findings, and facilitating comparisons with other studies (though these mental health conditions are more likely to co-occur). Like many longitudinal studies, the MCS experiences attrition (of the original 19,517 children, 10,757 attended the age 17 sweep) and missing data but the age 17 sample remains representative of the original cohort from the birth sweep. While all individuals had data on ethnicity and parental income, 4.3% were missing data on sexual identity and 3.5% homelessness. However, missing data did not substantially vary by homelessness. Missing data was addressed using the robust multiple imputation technique. While imputation relies on the MAR assumption which is not empirically verifiable [30], we increased the plausibility of the MAR assumption by including a rich set of auxiliary variables (maternal age at birth, parental mental health, mental wellbeing measures, all five SDQ subscales, BMI) in the imputation model. Auxiliary variables help increase the precision of predicting missing data and minimising non-random variation in values. Additionally, longitudinal data on BMI, high-quality socioeconomic measures and ethnicity are powerful predictors of missing data which significantly strengthen the imputation model.

A significant limitation is the small number of individuals who reported past experiences of homelessness which impacts statistical power especially in testing interactions effects by sexual identity sub-groups. However, this is severely impacted by individuals who experience homelessness are significantly under-represented in population-based studies. This necessitated combining bisexual, gay and lesbian individuals in the same category. However, despite small numbers, we still found significant findings for some sexual identity groups (e.g., bisexual individuals) and outcomes (e.g., self-harm, smoking, antisocial behaviour). Nonetheless, the smaller number of individuals in some categories resulted in reduced statistical power, affecting the precision of estimates in models including interactions (e.g., the wider CI related to past homelessness and gay/lesbian individuals and CIs for attempted suicide, depression, general health among sexual minority individuals with homelessness). As such, while these estimates are considered ‘statistically significant’, we advise caution in interpretation. The small number of sexual minority individuals with past homelessness precluded analyses of differences by sex, gender and ethnicity. There could be reporting bias in some outcomes like self-harm, attempted suicide and drug use as some individuals may not want to recall past distressing events, but we do not think this will vary between sexual identity or homeless groups. Associations with outcomes assessed over a shorter duration in the period just before the MCS age 17 sweep like sleep quality and exercise might impact reported findings. We could not distinguish between different types of homelessness (for example street-based vs. staying with other people), which might have differential associations with the outcomes assessed. There could also be recall bias in reporting past experiences of homelessness especially if it occurred at very young ages which can underestimate both the prevalence of past homelessness and associations with outcomes. The kind of homelessness experienced (e.g., staying with extended family and friends) may also impact reporting as young children may not understand this as experiencing homelessness. The wording of the question in the MCS age 17 survey on sex without contraception may not capture the full range of sexual activities across all sexual identities.

We were unable to examine the main causes of homelessness and whether these varied by sexual identity as these data were not collected. We could not examine the primary reasons for and period/length of homelessness, the kind of shelter sought, access to MH services, peer-group support, SM-specific issues such as gender-role strain, rejection sensitivity, internalised stigma, and perceived burdensomeness which are strongly linked to MH and well-being and also differ between individuals with and without past homelessness and sexual-identity groups. Due to small numbers, we were unable to examine associations in trans-, pansexual, and asexual individuals who have higher odds for homelessness compared to gay, lesbian and bisexual peers, [31]. As we are using one wave and a cross-sectional study design, we cannot conclude causality. Nonetheless, we examined associations between prior experience of homelessness (which could occur any time before age 17) and a range of outcomes assessed at age 17. Further, previous studies have found that past experiences of homelessness are subsequently associated with low mood/depression, risk for self-harm and attempted suicide in adolescents [8, 32].

### Comparison of findings with existing literature

To our knowledge, this is the first study to examine sexual identity related differences in past experiences of homelessness and associations with mental health and HRBs using a nationally representative sample of adolescents and robust statistical methods. Our study confirms previous findings that sexual minority adolescents are significantly more likely to experience homelessness compared to heterosexual peers [8, 33]. Most published studies combined sexual minority sub-groups together masking potential underlying differences between gay, lesbian and bisexual groups, an issue highlighted in systematic reviews [8, 33]. One of the largest studies to date including 28,405 sexual minority individuals did not report differences in homelessness by sexual minority subgroup [14]. However, some studies have found that bisexual youth experience higher rates of homelessness compared to gay and lesbian peers in line with our findings, others have reported no differences [31, 34]. Direct comparisons are limited as many studies did not include a heterosexual comparator group. This study also confirms higher rates of worse mental health and HRBs in sexual minority adolescents with past experiences of homelessness compared to sexual minority and heterosexual peers with and without past homelessness. We observed higher probabilities for worse mental health among sexual minority adolescents with past homelessness consistent with all indicators though statistically significant differences were limited to self-harm and attempted suicide. A key strength of our study is that we examined associations with multiple indicators of mental health in a probability sample, while most previous studies focused on depression and/or self-harm only. Similarly, we confirm previous findings on higher rates of HRBs among sexual minority adolescents with past experiences of homelessness with particularly stark differences for regular smoking and antisocial behaviour. To our knowledge this is the first study to examine antisocial behaviour in adolescents with past experiences of homelessness and differences by sexual identity.

A key finding was the statistically significant higher odds (and larger effect size compared to the other sexual minority subgroups) for past experiences of homelessness among bisexual individuals. Previous findings on higher risk of homelessness among bisexual youth compared to gay/lesbian peers are mixed and our finding could be due to better statistical power in group (6.5% of study participants identified as bisexual compared to 2.5% lesbian/gay) [34]. There is substantial evidence that bisexual individuals report worse health outcomes compared to gay/lesbian individuals with biphobia, stigma and social exclusion and rejection being common explanations [35]. While we were unable to examine mental health and HRBs in sexual minority subgroups, this needs to be examined in further detail using larger samples.

### Implications for public health

The lack of robust quantitative data on homelessness among sexual minority individuals and youth in particular, in the UK, has been repeatedly highlighted in recent reviews and reports on the topic [8, 33]. The only comprehensive government commissioned qualitative study and report on homelessness among LGBT individuals also highlighted the lack of quantitative studies in the UK and how it precludes further research and importantly prevents more holistic policy development [25]. Unfortunately, at time of writing, the current government has not indicated to prioritise adverse issues and their drivers, including homelessness experienced by the sexual and gender minority communities. As this study is the first in the UK to examine sexual identity inequalities in homelessness among youth using a nationally representative and probability sample of individuals, comparisons with other UK studies are limited.

This study takes the first step in addressing the lack of quantitative data on the subject but also highlights issues like the absence of key specific data required to comprehensively study homelessness in sexual minority individuals. Another issue highlighted is that current studies like the MCS, which despite being the largest of the UK birth cohort studies to date, are underpowered to study issues like homelessness in detail. Government policy needs to address this by establishing dedicated studies to examine health in marginalised groups including sexual minority and homeless populations. Current and new population-based studies could include oversampling marginalised groups to facilitate more robust studies on health in these groups. Our study’s findings and especially when examined together with the many qualitative studies and surveys conducted by UK-based charities and other stakeholders highlights that homelessness among sexual minority youth is a pertinent and ongoing issue and there are currently no government guidelines or agenda to address this. Family conflict, abuse and rejection due to sexual minority identity are the most common reasons for homelessness among sexual minority adolescents [8, 12]. This requires developing relevant and specific policies to help sexual minority individuals experiencing homelessness which are currently lacking in the UK. These could include more funding to better equip existing LGBTQ + homeless charities, establishing more shelters for sexual minority youth (especially in smaller towns and rural areas), and sensitising staff including relevant training in non-LGBTQ + homeless charities and shelters. Further, staff in schools, local community health centres (for example GPs) should also be made aware of the higher risk of homelessness in sexual minority individuals and the potential impact on their mental health. LGBTQ + relevant and appropriate counselling (currently inadequate) needs to be made more available in schools, the NHS and other places.

### Final summary

This study found higher prevalence of past experiences of homelessness among bisexual adolescents compared to heterosexual peers. Further, this study found a pattern indicating that sexual minority adolescents with past experiences of homelessness had higher prevalence of worse current mental health and some HRBs at age 17 compared to heterosexual peers with past experiences of homelessness. Importantly, this study is the first in the UK to quantitatively demonstrate these findings using a nationally representative sample. This study highlights the persistent and intersectional inequalities experienced by sexual minority youth which can further impact and widen health disparities across the lifecourse.

## Supplementary Information

Supplementary Material 1

## Data Availability

Data for this study is from the age 17 sweep of the Millennium Cohort Study. Data can be accessed from the UK Data Service website: [https://ukdataservice.ac.uk] (after registration and agreeing to license terms and conditions). Syntax/code used for the analysis in this study is available on request from the corresponding author.

## Abbreviations

EM: Ethnic minority
HRBs: : Health-risk behaviours
LGBTQ+: Lesbian, gay, bisexual, trans, queer and other sexual/gender minority identities
MCS: Millennium Cohort Study
OR: Odds ratio
SM: Sexual minority
SDQ-E: Strengths and difficulties questionnaire emotional symptoms subscale
