# Supplementary material for: Prevalence of past homelessness, mental health and health risk behaviours among sexual minority young people in the UK: insights from the Millennium Cohort Study
Source: BMC Public Health. 2025 Dec 10;25:4241. doi: 10.1186/s12889-025-25429-7 (PMC12696943; doi:10.1186/s12889-025-25429-7)
Supplement: Supplementary file 1 — Supplementary Material 1 [file 12889_2025_25429_MOESM1_ESM.docx]

**Prevalence of Past Homelessness, Mental Health and Health Risk Behaviours Among Sexual Minority Young People in the UK: Insights from the Millenium Cohort Study**

**Supplementary material**

**Supplemental Table 1. The original categories for sexual and ethnic-identity variables that were combined for analysis**

| **Original categories** | **N (%)** | **Collapsed for analysis** | **Final N (%)** |
| --- | --- | --- | --- |
| **Ethnicity** |  |  |  |
| White | 7,920 (81) | **White** | **7,920 (81)** |
| Mixed | 290 (3) |  |  |
| Indian | 287 (2.9) |  |  |
| Pakistani | 537 (5.5) |  |  |
| Bangladeshi | 243 (2.4) | **Ethnic minority** | **1,869 (19)** |
| Black Caribbean | 108 (1.1) |  |  |
| Black African | 219 (2.2) |  |  |
| ‘Other’ ethnic group | 185 (1.9) |  |  |
|  |  |  |  |
| **Sexual identity** |  |  | **Used in main analysis** |
| Completely heterosexual/ straight | 7,809 (78.5) | **Heterosexual** | **7,809 (89)** |
| Mainly heterosexual/ straight | 1,084 (10.9) |  |  |
| Bisexual | 649 (6.5) |  |  |
| Mainly gay or lesbian | 90 (0.9) | **Sexual minority** | **896 (11)** |
| Completely gay or lesbian | 157 (1.6) |  |  |
| Other (not included) | 154 (1.6) |  |  |
|  |  |  |  |

**Supplemental Table 2. A detailed description of health and wellbeing outcomes, and health-related behaviours assessed at the age 17 sweep of the Millennium Cohort Study**

| **Outcome** | **Question(s) in cohort member computer-assisted personal interview (CAPI), self-completion interview (CASI) or online questionnaire (CAWI)** | **Binary or continuous** | **Comments** |
| --- | --- | --- | --- |
| **Mental health and general health** |  |  |  |
| General health description | How would you describe your health generally? Would you say it is:  Excellent  Very good  Good  Fair  Poor | Excellent/very good/good vs fair/poor |  |
| Self-reported Strengths and Difficulties Questionnaire (SDQ)  -emotional subscale | Complains of headaches/stomach aches/sickness  Often seems worried  Often unhappy  Nervous or clingy in new situations  Many fears, easily scared. | Binary:  ‘close to average’ (<6) vs. ‘high/very high levels’ (≥6) of difficulties | 1. Not true  2. Somewhat true  3. Certainly true. |
| Attempted suicide | Have you ever hurt yourself on purpose in an attempt to end your life? | No vs yes |  |
| Doctor diagnosed depression | Has a doctor ever told you that you suffer from depression or serious anxiety? | No vs yes |  |
| Victimisation | In the past 12 months has anyone done any of these things to you?  Insulted/physical/hit/harassed/assaulted you. | No vs yes (any kind of victimisation) |  |
| Self-harm | During the last year, have you hurt yourself on purpose in any of the following ways?  Cut or stabbed yourself  Burned yourself  Bruised or pinched yourself  Taken an overdose of tablets  Pulled out your hair  Hurt yourself some other way | No vs yes (any kind of self-harm) |  |
| Sleep quality | During the past month, how would you rate your sleep quality overall? Would you say it has been…  1 …Very good  2 …Fairly good  3 …Fairly bad, or  4 …Very bad? | Very good/fairly good vs fairly bad/very bad |  |
| **Health and risk behaviours** |  |  |  |
| Frequency of alcohol consumption | How many times have you had an alcoholic drink in the last 12 months? | <10 times vs ≥10times |  |
| Frequency of cannabis use | In the past year how many times have you taken cannabis? | <4 times vs >4 times |  |
| Regular use of contraception) | Do you or any partner regularly use any of these forms of contraception or protection when having sex together?  Options:  Condom  The Pill  Implant  Injection  Emergency Contraception (e.g. morning after pill, emergency IUD/coil)  Some other form of contraception | No vs yes |  |
| Exercise in previous week | On how many days in the last week did you do a total of at least an hour of moderate to vigorous physical activity?  By moderate to vigorous we mean any physical activity that makes you get warmer, breathe harder and makes your heartbeat faster, e.g., riding a bike, running, playing football, swimming, dancing, etc. | None vs. any amount |  |
| Antisocial behaviour | Any antisocial behaviour in previous 12 months:  Pushed or shoved/hit/slapped/punched someone?  Hit someone with or used a weapon?  Stolen something from someone. e.g. a mobile phone, money etc.?  Harassed or bothered someone via mobile phone or email?  Sent pictures or spread rumours about someone via phone, email, social media or online?  Made an unwelcome sexual approach or assaulted someone sexually? | Yes or no |  |

**Supplemental Table 3. Associations between sexual identity and past experiences of homelessness in 10,232 adolescents aged 17 years from the Millennium Cohort Study. Estimates are from logistic regression models**.

| **Predictor** | **Unadjusted model** | | **Adjusted model^a^** | |
| --- | --- | --- | --- | --- |
|  | **OR** | **95% CI** | **OR** | **95% CI** |
| **Sexual identity** |  |  |  |  |
| Heterosexual | 1 |  | 1 |  |
| Mainly heterosexual | 1.04 | 0.48, 2.27 | 1.12 | 0.51, 2.49 |
| Bisexual | **3.09** | **1.59, 6.00** | **2.92** | **1.47, 5.82** |
| Gay/lesbian | 1.93 | 0.55, 6.75 | 1.87 | 0.53, 6.56 |
| **Sex** |  |  |  |  |
| Males |  |  | 1 |  |
| Females |  |  | 1.14 | 0.73,1.80 |
| **Parental income** |  |  |  |  |
| 1 (lowest quintile) |  |  | **6.60** | **3.38, 12.89** |
| 2 |  |  | **3.71** | **1.75, 7.87** |
| 3 |  |  | 1.35 | 0.58, 3.14 |
| 4/5 (highest quintiles) |  |  | 1 |  |
| **Ethnicity** |  |  |  |  |
| White |  |  |  |  |
| Ethnic minority |  |  | 0.78 | 0.43, 1.40 |

^a^Adjusted for sex assigned at birth, ethnicity and parental income.

Estimates in bold indicate 95% CIs that do not include 1.

**Supplemental Table 4. Associations between past homelessness and mental health in 10,223 adolescents aged 17 years from the Millennium Cohort Study**

|  | **SDQ symptoms subscale^a^** | | **Doctor diagnosed depression** | | **Self-harm^b^** | | **Attempted suicide^c^** | | **Poor general health^d^** | | **Poor sleep quality^e^** | |
| --- | --- | --- | --- | --- | --- | --- | --- | --- | --- | --- | --- | --- |
|  | **OR** | **95% CI** | **OR** | **95% CI** | **OR** | **95% CI** | **OR** | **95% CI** | **OR** | **95% CI** | **OR** | **95% CI** |
| **Past homelessness** |  |  |  |  |  |  |  |  |  |  |  |  |
| No | 1 |  | 1 |  | 1 |  | 1 |  | 1 |  | 1 |  |
| Yes | 3.24 | 1.91,5.51 | 4.45 | 2.19,9.07 | 2.90 | 1.54,5.44 | 5.26 | 2.49,11.14 | 4.75 | 2.38,9.49 | 3.17 | 1.37,7.37 |
| **Sexual identity** |  |  |  |  |  |  |  |  |  |  |  |  |
| Heterosexual | 1 |  | 1 |  | 1 |  | 1 |  | 1 |  | 1 |  |
| Mainly heterosexual | 1.92 | 1.60,2.31 | 1.69 | 1.34,2.13 | 2.50 | 2.11,2.96 | 1.83 | 1.37,2.45 | 1.79 | 1.37,2.34 | 1.44 | 1.21,1.72 |
| Bisexual/gay/lesbian | 3.06 | 2.60,3.59 | 3.39 | 2.82,4.07 | 5.67 | 4.67,6.89 | 4.05 | 3.12,5.24 | 2.14 | 1.65,2.77 | 1.82 | 1.54,2.16 |
| ***Interaction terms*** |  |  |  |  |  |  |  |  |  |  |  |  |
| homelessness **X** mainly heterosexual | 0.24 | 0.03,1.70 | 2.50 | 0.38,16.56 | 0.45 | 0.09,2.27 | 0.04 | 0.00,0.57 | 2.71 | 0.57,12.85 | 0.22 | 0.03,1.58 |
| homelessness **X** bisexual/gay/lesbian | 0.43 | 0.15,1.26 | 0.55 | 0.15,1.98 | 1.65 | 0.41,6.69 | 1.02 | 0.24,4.27 | 0.61 | 0.16,2.28 | 1.91 | 0.18,20.64 |
| **Sex at birth** |  |  |  |  |  |  |  |  |  |  |  |  |
| Males | 1 |  | 1 |  | 1 |  | 1 |  | 1 |  | 1 |  |
| Female | 3.54 | 3.10,4.04 | 2.15 | 1.83,2.53 | 1.63 | 1.43,1.85 | 2.07 | 1.68,2.55 | 1.16 | 0.96,1.39 | 1.1 | 0.98,1.24 |
| **Ethnicity** |  |  |  |  |  |  |  |  |  |  |  |  |
| White | 1 |  | 1 |  | 1 |  | 1 |  | 1 |  | 1 |  |
| Ethnic-minority | 0.58 | 0.48,0.70 | 0.43 | 0.32,0.56 | 0.64 | 0.52,0.78 | 0.6 | 0.46,0.78 | 1.01 | 0.80,1.29 | 0.93 | 0.78,1.11 |
| **Parental income** |  |  |  |  |  |  |  |  |  |  |  |  |
| Quintile 1 | 1.22 | 1.03,1.45 | 1.76 | 1.42,2.19 | 1.27 | 1.06,1.51 | 2.75 | 2.15,3.52 | 2.51 | 1.97,3.21 | 1.36 | 1.13,1.64 |
| Quintile 2 | 1.24 | 1.05,1.46 | 1.45 | 1.16,1.81 | 1.24 | 1.06,1.45 | 2.18 | 1.69,2.82 | 1.67 | 1.28,2.16 | 1.31 | 1.12,1.54 |
| Quintile 3 | 1.23 | 1.06,1.44 | 1.42 | 1.16,1.74 | 1.05 | 0.92,1.20 | 1.35 | 1.00,1.81 | 1.37 | 1.01,1.85 | 1.25 | 1.07,1.48 |
| Quintiles 4 & 5 (most advantaged) | 1 |  | 1 |  | 1 |  | 1 |  | 1 |  | 1 |  |

SDQ: Strengths & difficulties questionnaire. ^a^Those with scores ≥6 (high/very high levels of emotional difficulties), ^b^self-harm in previous year, ^c^lifetime attempted suicide, ^d^current general health, ^e^sleep quality in previous month

**Supplemental Table 5. Associations between past homelessness and health-risk behaviours in 10,223 adolescents aged 17 years from the Millennium Cohort Study**

|  | **Alcohol frequency (≥10 times^a^)** | | **Cannabis frequency**  **(≥5 times^a^)** | | **Current regular smoking** | | **No exercise^b^** | | **Sex without contraception^c^** | | **Anti-social behaviour^a^** | |
| --- | --- | --- | --- | --- | --- | --- | --- | --- | --- | --- | --- | --- |
| **Past homelessness** | **OR** | **95% CI** | **OR** | **95% CI** | **OR** | **95% CI** | **OR** | **95% CI** | **OR** | **95% CI** | **OR** | **95% CI** |
| No | 1 |  | 1 |  | 1 |  | 1 |  | 1 |  | 1 |  |
| Yes | 0.41 | 0.16,1.09 | 1.45 | 0.58,3.61 | 3.11 | 1.53,6.32 | 1.42 | 0.75,2.68 | 2.13 | 1.18,3.83 | 0.92 | 0.47,1.79 |
| **Sexual identity** |  |  |  |  |  |  |  |  |  |  |  |  |
| Heterosexual | 1 |  | 1 |  | 1 |  | 1 |  | 1 |  | 1 |  |
| Mainly heterosexual | 1.16 | 0.98,1.38 | 1.59 | 1.23,2.06 | 1 | 0.79,1.27 | 1.1 | 0.92,1.32 | 1.11 | 0.90,1.36 | 1.4 | 1.17,1.69 |
| Bisexual/gay/lesbian | 1.06 | 0.87,1.30 | 1.66 | 1.27,2.18 | 1.60 | 1.28,2.00 | 1.53 | 1.25,1.86 | 1.32 | 1.09,1.59 | 1.27 | 1.06,1.53 |
| ***Interaction terms*** |  |  |  |  |  |  |  |  |  |  |  |  |
| homelessness **X** mainly heterosexual | 4.54 | 0.74,27.98 | 1.85 | 0.17,20.52 | 0.61 | 0.11,3.39 | 0.29 | 0.05,1.60 | 1.96 | 0.46,8.31 | 5.13 | 1.18,22.26 |
| homelessness **X** bisexual/gay/lesbian | 4.64 | 0.89,24.11 | 2.01 | 0.39,10.48 | 1.72 | 0.48,6.09 | 0.65 | 0.19,2.16 | 1.09 | 0.30,4.02 | 4.03 | 1.23,13.19 |
| **Sex at birth** |  |  |  |  |  |  |  |  |  |  |  |  |
| Males | 1 |  | 1 |  | 1 |  | 1 |  | 1 |  | 1 |  |
| Female | 0.80 | 0.71,0.89 | 0.41 | 0.34,0.49 | 0.94 | 0.81,1.10 | 1.85 | 1.66,2.06 | 1.02 | 0.90,1.17 | 0.34 | 0.30,0.38 |
| **Ethnicity** |  |  |  |  |  |  |  |  |  |  |  |  |
| White | 1 |  | 1 |  | 1 |  | 1 |  | 1 |  | 1 |  |
| Ethnic-minority | 0.26 | 0.19,0.36 | 0.71 | 0.53,0.95 | 0.27 | 0.20,0.36 | 1.14 | 0.96,1.34 | 0.36 | 0.27,0.47 | 1.09 | 0.93,1.27 |
| **Parental income** |  |  |  |  |  |  |  |  |  |  |  |  |
| Quintile 1 | 0.40 | 0.33,0.48 | 1.06 | 0.81,1.40 | 2.89 | 2.37,3.53 | 1.89 | 1.61,2.23 | 1.32 | 1.10,1.58 | 1.02 | 0.86,1.22 |
| Quintile 2 | 0.59 | 0.49,0.71 | 1.17 | 0.91,1.50 | 2.23 | 1.83,2.72 | 1.75 | 1.49,2.06 | 1.24 | 1.06,1.45 | 1.03 | 0.88,1.21 |
| Quintile 3 | 0.70 | 0.60,0.82 | 0.98 | 0.79,1.22 | 1.34 | 1.08,1.65 | 1.35 | 1.14,1.60 | 1.19 | 1.02,1.39 | 0.92 | 0.79,1.07 |
| Quintiles 4 & 5 (most advantaged) | 1 |  | 1 |  | 1 |  | 1 |  | 1 |  | 1 |  |

^a^in previous 12 months, ^b^in previous week, ^c^regular use of contraception or protection.
